# Solitary Head and Neck Cysticercosis: A Series of Rare Cases

**Published:** 2017-11

**Authors:** Priyanko Chakraborty, Rakhi Kumari, Rajiv-Kumar Jain, Vikash Prasad, Sidharth Pradhan, Purnima Joshi

**Affiliations:** 1 *Department of Otorhinolaryngology, Institute Of Medical Sciences, Banaras Hindu University, Varanasi-221005.*; 2 *Department of Pathology, Institute of Medical Sciences, Banaras Hindu University, Varanasi-221005.*

**Keywords:** Albendazole, Cysticercosis, Cytopathology, Soft Tissue Cysticercosis

## Abstract

**Introduction:**

Cysticercosis is a disease which is caused by the infestation of the larvae *Taenia solium*, with humans acting as an intermediate host instead of a definitive host. Head and neck involvement including maxillofacial and oral involvement of cysticercosis is quite rare.

**Case Report:**

We report a series of rare cases of cysticercosis of the head and neck region encountered in a tertiary hospital in Northern India with a brief review of literature and its diagnosis and management. The patients had undergone ultrasonography, FNAC and CT scan. All the cases were treated by Oral Albendazole tablets. The period of study was from August 2014 to August 2015. FNAC proved to be a highly effective way of diagnosis corroborated by imaging evidence. Treatment with albendazole was curative in all the cases.

**Conclusions:**

Cytopathology has emerged as an excellent diagnostic modality for cysticercosis. Medical treatment with antihelminthics produces excellent results, as illustrated in our case, and can eliminate the need of surgery.

## Introduction

 Cysticercosis is an infestation of the larva Cysticercus cellulosae, of *Taenia solium*. It is the pork tapeworm belonging to the phylum Platyhelminthes which is a cyclophyllid cestode in the family Taeniidae.

Platyhelminthes have a two staged life cycle; first as a larva which develops from egg and then as an adult worm. Each of these phases requires a different host. Humans usually acts as the definitive host and harbor the adult stage, intestinal Taenia solium. The parasitic infestation by the pork tapeworm larval stage, cysticercosis, is caused by the ingestion of tapeworm eggs through contaminated food, water or dirty hands. In this case man acts as the intermediate host (1).

 Cysticercosis, being a rare entity is seldom suspected for an isolated swelling over the head and neck region thus leading to a diagnostic dilemma for clinicians. 

## Case Report

The patients who attended the otolaryngology outpatient department of our hospital from August 2014 to August 2015 were included. Four cases of isolated head and neck cysticercosis were reported. 

*Case 1:*

**Fig 1:**
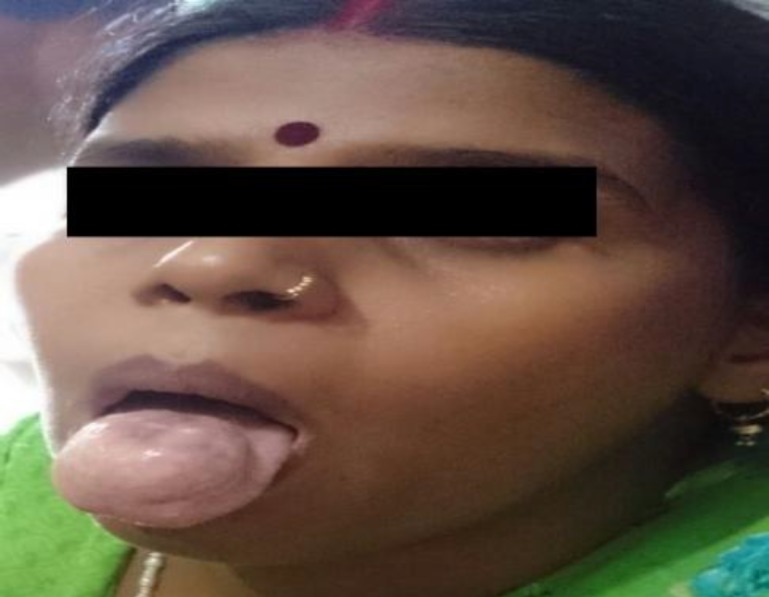
Globular swelling dorsum of tongue.

A 27-year-old woman presented with the chief complaint of a painless nodule on the dorsum of her tongue for the past six months, with gradual increase in size to approximately 4x4 cm. Examination revealed a tense cystic, nontender nodule that was covered with normal mucosa without any fixity (Fig.1). 

Fine needle aspiration cytology from the swelling pointed towards the diagnosis of cysticercosis. A CT scan of the oral cavity demonstrated a cystic lesion of the tongue. It did not show any calcification or definite scolex. The patient was recommended for surgery; but a trial of Albendazole tablets was given in an attempt to reduce the vascularity and size. Surprisingly the lesion subsided completely after the course of Albendazole thereby eliminating the need of surgery. The patient is under regular follow up with no recurrence of the swelling. 

*Case 2:*

**Fig 2:**
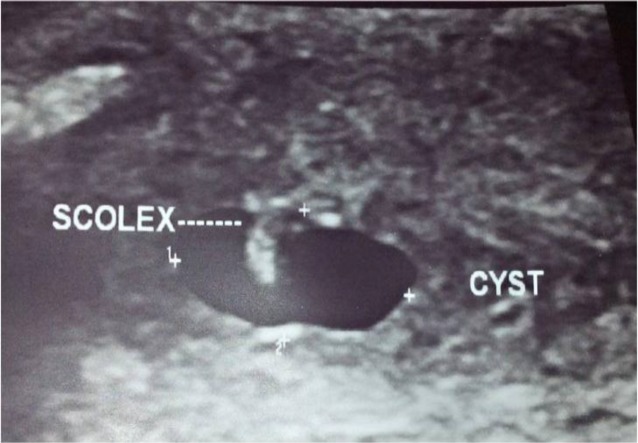
Ultrasound tracing of neck showing the features of a definite cystic lesion with a scolex within it

A 33-year-old man presented with a painless progressive swelling over the left anterior neck for a duration of four months. During examination a non-tender, tense, cystic swelling was palpable deep in the sternocleidomastoid muscle. Ultrasonography of the neck showed the features of a definite cystic lesion with a scolex within it (Fig.2). CT scan of the neck also supported the findings. FNAC confirmed it to be a case of cysticercosis of the sternocleidomastoid muscle. The patient was treated with Albendazole tablets.

*Case 3*

 A 28-year-old woman presented with a painless swelling below the chin for 8 months. She was treated with ATT empirically in another institution for 7 months without resolution. Upon examination a firm globular, non-tender lump was palpated in the submental region. We did an ultrasonography of the neck and FNAC from the lump and finally reached to the diagnosis of cysticercosis of the floor of the mouth. Albendazole tablets were given as the treatment.

*Case 4*

**Fig 3:**
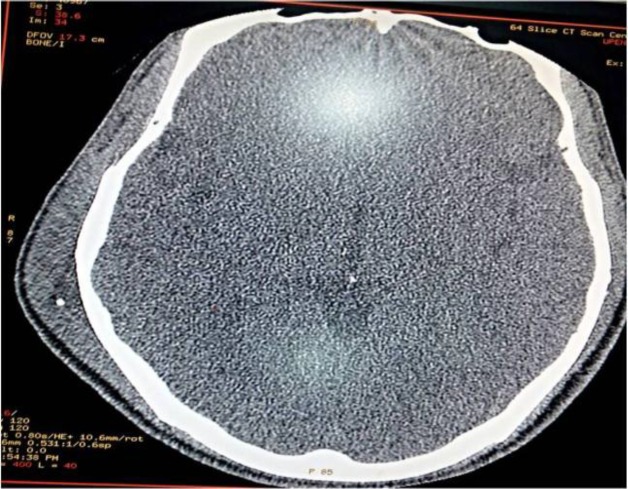
CT scan of the temporal bone showing unifocal calcification on the right side within the substance of the temporalis muscle with swelling.

 A 35-year-old male presented with a gradually progressive hard, mildly painful swelling over the right temporal bone for a period of 1 year. Upon examination a non-tender bony hard swelling over the right temporal bone was palpated. The patient underwent a CT scan of the temporal bone which showed unifocal calcification within the temporalis muscle (Fig.3). 

FNAC initially yielded bloody tap. A repeat FNAC performed a week later; however, supported the diagnosis of cysticercosis. The patient was treated with Albendazole and the swelling went down. 

All the patients were given a standard course of Tablet Albendazole with a dosage of 15mg/kg per day or standard adult dose of 400mg per day for 28 days. CT scan of the brain was performed prior to starting the Albendazole therapy to rule out neurocysticercosis.

**Fig 4:**
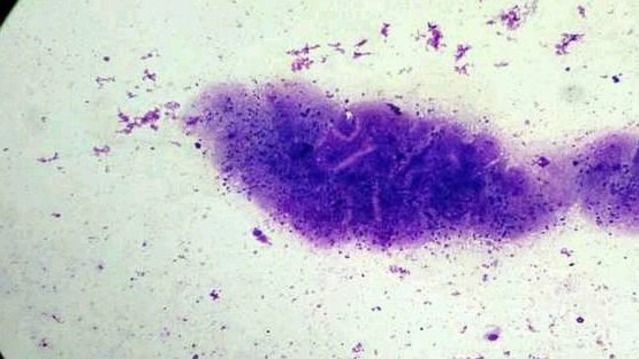
Cytology (Giemsa stain, 100X): Bluish fibrillary fragments with areas of honeycomb appearance and hyaline membrane surrounding it with varying grades of mixed inflammatory infiltrates.

Smears from the straw colored clear fluid obtained by fine needle aspiration from the swellings showed fibrillary fragments, bluish in color, which were interspersed with small nuclei. Areas of honeycomb appearance were also seen. The background demonstrated mixed inflammatory cells comprising of neutrophils, eosinophils, histiocytes and lymphocytes with occasional granulomas (Fig.4). Giemsa staining showed that the outer cuticular matrix stained lightly, while the inner cellular layer staining darkly. This pointed toward the diagnosis of cysticercosis. Microscopy of stool did not reveal any Taenia eggs in any patient. Complete blood count was normal in all patients. Absolute eosinophil count ranged between 140 and 600 eosinophils per cubic millimeter. 

The patients are under regular follow up with no recurrence of the swelling. Cranial CT scan and indirect ophthalmoscopy was performed in all cases with none of the cases showing any abnormalities.

## Discussion

Human cysticercosis is almost always caused by Cysticercus cellulosae (2). According to some authors cysticercosis produced by Cysticercus bovis (Taenia saginata) is unknown in humans (3). Humans are the definitive host of the adult form of the Taenia solium tape worm and pigs are the intermediate host of the larval stage. However, humans can become an accidental intermediate host of T*. *solium when viable eggs of the parasite are ingested. The eggs of the parasite are often present in unwashed and undercooked vegetables and salads. The eggs hatch in the digestive tract and the larvae are transported through the bloodstream to their destination, which is generally the central nervous system, muscles and subcutaneous tissues (4,5,6). Cases of cysticercosis involving oral tissues are rare but may affect the tongue, lower lip and oral mucosa, in this order of prevalence (4,7,8). 

The diagnostic role of FNAC in cysticercosis was first emphasized by Kung et al. in 1989(9). Saran et al., proposed the use of fine needle aspiration cytology, which identifies the tegument layer of the larva, to help the clinician in planning the treatment (10). Essential for the cytodiagnosis of cysticercosis is the identification of the parasitic fragments including its bladder wall and hooklets (11). Parasitic fragments may comprise bluish, fibrillary structures, sometimes with honey- combing, calcospherules, tegument thrown into rounded wavy folds, scolex with hooklets, and hyaline membrane surrounding it with varying grades of mixed inflammatory infiltrates (11,12,13). Finding an entire scolex in FNAC is a rare event (14).

USG is a, non-invasive, non-ionizing, sensitive modality for diagnosing cysticer- cosis. Four different sonographic patterns of muscular cysticercosis have been described. 

The first type is cysticercus cyst with an inflammatory mass around it, caused by the death of the larva. The second type is an irregular cyst with very minimal fluid on one side, indicating a leakage of fluid. The third appearance is a large irregular collection of exudative fluid within the muscle with the typical cysticercus cyst containing the scolex, situated eccentrically within the collection. The fourth sonographic appearance is that of calcified cysticercosis (15).

All of our cases were provisionally diagnosed as cystic lesion of the respective site, with cysticercosis late in the list of differentials. However, characteristic imaging findings supported by positive cytology led to the final diagnosis of cysticercosis. This underlines the need for a high index of suspicion during diagnosis and also emphasizes the incidence of cysticercosis in the developing world. In spite of the high prevalence of cysticercosis in some parts of the world, oral and perioral lesions are relatively rare. The most common locations of the reported cases are shown in (Table.1) (11,12,14,16). 

**Table 1:** Case reports of oral cysticercosis

**Site of involvement**	**Number of cases**
Tongue	44
Lip	27
Buccal Mucosa	24
Other sites (gingiva, floor of mouth, retro molar, submental, subcutaneous mandible, neck midline and soft palate)	12

Pubmed database for the last two decades was reviewed in which only 3 cases of cysticercosis involving the temporalis muscle were found to be documented (7,10,16).Our case of tongue cysticercosis is the second case encountered in our institute in a period of more than 25 years. The previous one, reported in 1989, depicts the rarity of tongue involvement (6). 

Every case of head and neck cysticercosis should be thoroughly investigated to determine the involvement of multiple sites, as there is high incidence of multilocularity Drugs such as praziquantel (50-100 mg/kg three divided doses for 15-30 days) and Albendazole (15mg/kg per day for 8-28 days) are potent antihelminthics used in the treatment of cysticercosis (2). Oral steroids are recom- mended along with cysticidal drugs to control the inflammation elicited by the dying cyst in cases of neurocysticercosis or ocular cysticercosis to prevent concomitant damage to delicate vital structures (17). CT scan of the brain and indirect ophthalmoscopy is recommended before the start of cysticidal drugs in all cases of cysticercosis for the same reason (17). Cases of solitary cysticercosis nodules which are easily accessible are usually excised surgically (5,6,7,16). However, in our case series, only antihelminthic Albendazole tablets, administered over a period of 28 days, produced excellent results with complete regression of the cyst in all of the cases. 

Albendazole causes degenerative alterations in the intestinal cells of the worm by binding to the colchicine-sensitive site of tubulin, thus inhibiting its polymerization or assembly into microtubules. The loss of the cytoplasmic microtubules leads to impaired uptake of glucose by the larval and adult stages of the susceptible parasites and depletes their glycogen stores. Degenerative changes occur in the endoplasmic reticulum, the mitochondria of the germinal layer, and the subsequent release of lysosome (2).

## Conclusion

Cysticercosis still remains one of the elusive diseases for clinicians to diagnose. Medical treatment with antihelminthics produces excellent results, as illustrated in our case, and can eliminates the need of surgery.
